# Potential Protective Effects of Antioxidants against Cyclophosphamide-Induced Nephrotoxicity

**DOI:** 10.1155/2022/5096825

**Published:** 2022-04-16

**Authors:** Muluken Altaye Ayza, Kaleab Alemayehu Zewdie, Elias Fitsum Yigzaw, Solomon Gashaw Ayele, Bekalu Amare Tesfaye, Gebrehiwot Gebremedhin Tafere, Muzey Gebreyohannes Abrha

**Affiliations:** ^1^Department of Pharmacology, School of Pharmacy, College of Medicine and Health Sciences, Hawassa University, Hawassa, Ethiopia; ^2^Department of Pharmacology and Toxicology, School of Pharmacy, Mekelle University, Mekelle, Ethiopia; ^3^Clinical Pharmacy Unit, School of Pharmacy, College of Health Sciences, Mekelle University, Mekelle, Ethiopia; ^4^Department of Pharmaceutics, School of Pharmacy, College of Health Sciences, Mekelle University, Mekelle, Ethiopia; ^5^Department of Medicinal Chemistry, School of Pharmacy, Mekelle University, Mekelle, Ethiopia

## Abstract

Cyclophosphamide is an alkylating antineoplastic agent, and it is one of the most successful drugs with wide arrays of clinical activity. It has been in use for several types of cancer treatments and as an immunosuppressive agent for the management of autoimmune and immune-mediated diseases. Nowadays, its clinical use is limited due to various toxicities, including nephrotoxicity. Even though the mechanisms are not well understood, cyclophosphamide-induced nephrotoxicity is reported to be mediated through oxidative stress. This review focuses on the potential role of natural and plant-derived antioxidants in preventing cyclophosphamide-induced nephrotoxicity.

## 1. Introduction

Cyclophosphamide is an oxazaphosphorine, alkylating agent of the nitrogen mustard class with potent cytotoxic and immunosuppressive effects; first synthesized by Arnold and colleagues in 1958 [1–3]. It is widely used in the treatment of malignant tumors and autoimmune diseases including lupus, systemic sclerosis, and several types of vasculitides [4].

**Figure 1:**
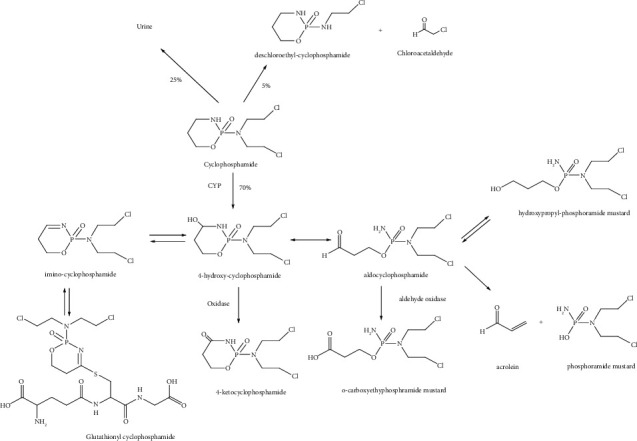
Major metabolism and disposition pathways of cyclophosphamide.

Cyclophosphamide is a biologically inactive prodrug that needs cytochrome-P450 mediated activation [5, 6]. The drug is first transformed into hydroxylated intermediates, which undergo breakdown to form the active compounds; phosphoramide mustard and acrolein (Figure 1). The final result of the reaction between phosphoramide mustard and DNA is cell death [7]. Another metabolite excreted in the urine is chloroacetaldehyde, which is formed during detoxification of cyclophosphamide. Acrolein and chloroacetaldehyde have been linked to various toxicities [8].

Cyclophosphamide is associated with various toxicities; with the cumulative dose being the principal risk factor [9]. Short-term effects include gastrointestinal effects (nausea, vomiting, anorexia, and diarrhea), myelosuppression, infection, alopecia, and hemorrhagic cystitis. Long-term risks include gonadal toxicity, teratogenicity, cardiotoxicity, and pulmonary toxicity [4, 9]. In addition to these, nephrotoxicity and hepatotoxicity can occur due to cyclophosphamide [10]. These multiple organ toxicities limited the clinical application of cyclophosphamide. One of the limiting effects of cyclophosphamide use is nephrotoxicity. Therefore, successful prevention of renal injury requires knowledge of pathogenic mechanisms and early interventions. This review focuses on the mechanisms and targets to ameliorate cyclophosphamide-induced nephrotoxicity.

## 2. Mechanisms and Role of Antioxidants in Cyclophosphamide-Induced Nephrotoxicity

Drugs are a common cause of kidney damage and contribute to morbidity and increased healthcare utilization [11, 12]. In patients with normal kidney function, nephrotoxicity can be considered if there is an increment in serum creatinine by ≥0.3 mg/dL within 48 h, increase in serum creatinine to ≥1.5 times baseline, which is known or presumed to have occurred within the prior 7 days, and/or urine volume <0.5 mL/kg/h for 6 h [13].

Even though the mechanisms are not completely understood, one of the possible mechanisms of drug-induced nephrotoxicity is oxidative stress [14–16]. Oxidative stress occurs when there is excess oxygen radicals produced in cells, which could overpower the normal antioxidant capacity [17]. During cyclophosphamide metabolism and degradation of its metabolites, there is generation of reactive oxygen species (ROS) encompassing superoxide anions, hydroxyl radicals, and hydrogen peroxide [18]. The production of free radicals in turn leads to disruption of several signaling pathways including the inflammation pathways, which can lead to organ fibrosis [19]. In response to oxidative stress, c-Jun N-terminal kinase pathway is activated, which in turn phosphorylates c-Jun. Then, c-Jun induces transcription of genes that mediate inflammatory response like tumor necrosis factor alpha (TNF-a). The activation of JNK/c-Jun pathway by exposure to toxic agents/drugs or cellular stresses led to renal fibrosis through stimulation of both inflammation and apoptosis [20].

Levels of superoxide dismutase-1 (Cu/Zn-dependent enzyme) expression play an important role in cyclophosphamide-induced nephrotoxicity [21]. This is supported by the finding that the activity of the superoxide dismutase (SOD) was significantly reduced in the rat kidneys treated with cyclophosphamide. In addition, protein nitration, nitrotyrosine, and poly (ADP-ribose) polymerase activation and NAD depletion may play a critical role in the pathogenesis of cyclophosphamide-induced kidney damage [22].

Furthermore, the activities of antioxidant enzymes including, SOD, catalase (CAT), glutathione peroxidase (GSH-Px), and glutathione reductase are decreased after cyclophosphamide administration in rats. Treatment with amifostine, an organic thiophosphate compound, significantly protected kidney antioxidant parameters from changes induced by cyclophosphamide and, as a result, it prevented oxidative stress and peroxidative damage [18].

When cells are exposed to hydroxy-cyclophosphamide, a form of cyclophosphamide induces lipid peroxidation [23]. As reported by [24], rats treated with cyclophosphamide showed high biochemical parameters such as malondialdehyde (MDA) and low antioxidant activity (GSH) compared to the control group. Pretreatment with seleno L-methionine, an antioxidant compound, significantly prevented cyclophosphamide-induced lipid peroxidation in the kidney tissues [24].

Furthermore, treatment with gallic acid restored the enzymic and nonenzymic antioxidants and also attenuated cyclophosphamide-induced nephrotoxicity through free radical scavenging activity, anti-inflammatory, and improvement of antioxidant defense system [25]. Other compounds/extracts also prevented cyclophosphamide-induced kidney damage, including ozone therapy [26], hydroalcoholic extract of *Capparis spinosa* [27], methanolic extract of *Curcuma caesia* [28], Diallyl sulfide [29], *Nigella sativa* oil, and thymoquinone [30] (Table 1). Additionally, currently available drugs also show renoprotective effect (Table 2). These findings support the association between oxidative stress and cyclophosphamide-induced nephrotoxicity.

Even though, various studies reported the association between cyclophosphamide-induced nephrotoxicity and oxidative stress, [71] showed that pretreatment with glutamine (the precursor for glutathione synthesis) did not prevent cyclophosphamide-induced lipid peroxidation and renal damage. This might indicate the involvement of mechanisms other than oxidative stress, and/or oxidative stress might be the consequence and not the cause of cyclophosphamide-induced renal damage [71]. Cyclophosphamide-induced nephrotoxicity might be related to energy metabolism, amino acid metabolism, choline metabolism, and nucleotide metabolism. The nuclear magnetic resonance-based metabolomics approach revealed cyclophosphamide administration elevated choline and creatine levels, this might be due to inhibited choline metabolism. Hypoxanthine level was also decreased and this might be due to cyclophosphamide-induced disturbance in purine metabolism, which can imply the impairment of renal function [72].

## 3. Novel Clinical Biomarkers of Kidney Injury

Nephrotoxicity is one of the major reasons that drugs are withdrawn from the market, and it is a major concern to drug approval agencies and manufacturing companies [73]. Some of the currently available drugs possess nephrotoxic properties and traditionally been detected and defined by reduced urine output and elevated serum creatinine concentrations [74]. Serum creatinine, blood urea nitrogen (BUN), and urine output are standard measures of nephrotoxicity [75].

Serum biomarkers like serum creatinine and BUN may not be adequate to accurately detect kidney injury. They are suboptimal, because they merely reflect changes in the glomerular filtration rate; which is a nonspecific measure of proximal tubular injury, and it becomes apparent only after significant kidney damage. These biomarkers are insensitive, and they may allow drugs to pass preclinical safety criteria only to be found nephrotoxic in patients during clinical trials or postmarket clinical use. Additional noninvasive urinary biomarkers, like kidney injury molecule-1 (KIM-1) and neutrophil gelatinase-associated lipocalin (NGAL) might be important [76–78].

KIM-1 is a phosphatidylserine receptor on renal epithelial cells that recognize apoptotic cells, directing them to lysosomes and thereby converting the normal proximal tubule cell into a phagocyte. KIM-1 mRNA levels are elevated after initiation of kidney injury and outperformed serum creatinine, BUN, and urinary N-acetyl-*β*-D-glucosaminidase in rat models of kidney injury [79]. Unlike other kidney injury markers, urinary KIM-1 was found elevated at the early stages of the kidney injury in rats, and these elevations were sustained throughout the progression of cell injury to proliferation and regeneration [78].

Furthermore, fifteen female breast cancer patients, after treatment with adriamycin and cyclophosphamide showed a significant increase in KIM-1 level. Urinary KIM-1 can be a sensitive biomarker for the evaluation of early kidney injury in cancer patients on chemotherapy [80]. Human KIM-1, a soluble form, can be detected in the urine of patients with acute tubular necrosis, and it can be a useful biomarker for renal proximal tubule injury and facilitating early diagnosis [81].

Neutrophil gelatinase-associated lipocalin (NGAL) is a 25-kDa protein, which is originally isolated from neutrophil secondary granules. It is freely filtered at the glomerulus and reabsorbed in the proximal tubule almost completely. Increased urinary excretion suggests proximal tubular damage with impaired reabsorption or increased primary synthesis and excretion by distal nephron segments [82]. It is one of the most promising renal markers [83]. In mice treated with cisplatin, NGAL is easily detected in the urine by western analysis within 3 hours of cisplatin administration in a dose and duration-dependent manner. But changes in urinary N-acetyl-*β*-D-glucosaminidase (NAG) or serum creatinine were not evident until 96 hours after cisplatin administration. NGAL can be an early and quantitative urinary biomarker for nephrotoxicity, and the most promising biomarker in clinical nephrology [84, 85].

Cyclophosphamide-induced cystitis with the tubulopathy character is expressed by the increased production and release of a fatty-acid binding protein (FABP) and osteopontin into the urine as markers reflecting acute kidney injury in rats [86]. FABP-4, predominantly expressed in adipocytes and macrophages, is increased in the urine of patients with a glomerular injury. It can have a potential value in the detection of early region-specific drug-induced kidney injury [87]. The level of FABP will increase after 4 hours of insult [82].

Another biomarker will be interleukin-18 (IL-18). It is a proinflammatory cytokine with 18 kDa molecular weight. One of the major sources of IL-18 production is a renal tubular cell. IL-18 is upregulated and increased during acute kidney injury [88]. It has been reported that IL-18 can be a promising marker of chronic renal injury in children after chemotherapy [89].

## 4. Hopes in the Management of Chemotherapy-Induced Nephrotoxicity

It has been reported that oxidative stress could be a key mechanism in cyclophosphamide-induced nephrotoxicity [51]. Thus, agents that can enhance host antioxidant defense mechanisms can be a promising target to ameliorate cyclophosphamide-induced nephrotoxicity [44]. Several studies reported that, enhancing the antioxidant and anti-inflammatory system can prevent nephrotoxicity (Table 1).

A study done by El-Shabrawy et al. [70] revealed that, tovaptan coadministration with cyclophosphamide significantly reduced lipid peroxidation marker, MDA, and proinflammatory cytokines. Due to tolvaptan coadministration, histopathological results also showed improvements of nephrotoxicity signs. As compared to cyclophosphamide-treated group, tolvaptan cotreatment significantly decreased the level of apoptosis markers as caspase-3 and Bax with increased expression of antiapoptotic Bcl-2 in renal tissue [70]. In addition, tolvaptan showed renoprotective effects in a Dahl hypertensive heart failure rats. In this study, renal fibrosis was found to be significantly inhibited in the tolvaptan-treated group of animals [90]. It can be concluded that, long-term therapy with tolvaptan may improve oxidative stress-induced renal dysfunction, podocyte injury, glomerulosclerosis, and inflammation [91].

A Chinese herbal complex Huaiqihuang, composed of *Trametes robiniophila* (Auriculariacaee), *Lycium barbarum* (Solanaceae), and *Polygonatum sibiricum* (Liliaceae) showed significant antioxidant, antiapoptosis, and anti-inflammatory activity and exhibits a therapeutic effect against renal diseases. Huaiqihuang showed protective effect against cyclophosphamide-induced nephrotoxicity in rats through decreasing the production of MDA, and increasing the activities of antioxidant enzymes including, CAT and SOD [92, 93].

An isoquinoline derivative alkaloid called Berberine proved to have an antioxidant and anti-inflammatory activity. Berberine showed protective effect against cyclophosphamide-induced renal injury through decreasing the level of kidney injury markers including, blood urea nitrogen, creatinine, kidney injury molecule-1 and increasing the level of antioxidants such as glutathione peroxidase, glutathione, superoxide dismutase, and catalase activities [94].

Through its antioxidant, antiapoptotic, and anti-inflammatory properties, berberine also exerted nephrprotective effects against gentamicin-induced nephrotoxicity in rats [95]. Berberine also resulted in an ameliorative effect against cisplatin-induced nephrotoxicity in rats, through enhancing the antioxidant capacity and reducing the oxidative stress markers in the renal tissue [96]. It can be concluded that, berberine might be a good candidate for nephroprotective effect and further studies need to be recommended.

Furthermore, in a randomized control trial, advanced ovarian cancer patients pretreated with amifostine revealed reduced cumulative hematologic, renal, and neurologic toxicities associated with the cyclophosphamide-regimen, with no reduction in antitumor efficacy [97]. Amifostine (ethyol or WR2721) is an FDA-approved sulfhydryl compound that is administered to patients as a cytoprotective treatment before chemotherapy. It protects normal cells by scavenging free radicals and through hydrogen donation to reactive oxygen species and regulating the transcription of genes involved in apoptosis, cell cycle, and DNA repair [98, 99]. In addition, amifostine resulted in the inhibition of oxidative stress and lipid peroxidation in the kidneys of cyclophosphamide-treated rats. Collectively, amifostine is reported to prevent cyclophosphamide-induced renal injury and dysfunction [18]. These and other antioxidants have the ability to diminish free radical formation and promote endogenous antioxidant enzyme activity; they can be a possible source for nephroprotective agents in the future.

## 5. Conclusion

Cyclophosphamide is an alkylating agent widely used for the treatment of cancer, and it has been used to treat severe manifestations of autoimmune inflammatory diseases, including systemic vasculitis, systemic lupus erythematosus, and systemic sclerosis. However, cyclophosphamide is associated with renal dysfunction, and its clinical use is limited due to various toxicities. Several natural and plant-based antioxidants have shown an important and promising nephroprotective activity in preclinical studies, and they might be an effective source for nephroprotctive agents. In addition to oxidative stress, there might be an involvement of other mechanisms related with energy, amino acid, choline, and nucleotide metabolism pathwys; worth considering. Further investigations are recommended to understand their safety and efficacy profile on a clinical basis. Translational clinical researches should be considered on those found to be safe and effective in preclinical studies.

## Figures and Tables

**Table 1:** Protective role of antioxidants against cyclophosphamide-induced nephrotoxicity.

References	Animal Used	Method and Intervention	Major Findings
[31]	Male Wistar rats (*n* = 35)	Rats were pretreated with naringin (NG) (50 and 100 mg/kg/day), for 7 days. Then, single dose of CP (200 mg/kg b.w.) was administering on the seventh day.	(i) ↓ levels of serum urea and creatinine (ii) ↓ levels of renal iNOS, COX-2, LC3B and significantly modulated renal NF-*κ*B, TNF-*α*, IL-1*β*, and IL-6 (iii) ↑ levels of renal SOD, CAT, GPx, GSH (iv) The protective effects of NG is possibly due to the mitigation of apoptosis, autophagy, inflammation, oxidative stress, and oxidative DNA damage.
[32]	Male Swiss albino mice (*n* = 25)	The mice were pretreated with ellagic acid (EA), orally at a dose of 50 and 100 mg/kg for 7 consecutive days before the administration of a single intraperitoneal (i.p.) injection of CP at 50 mg/kg.	(i) EA pretreatment restored the activities of antioxidant enzymes (ii) ↓ micronuclei formation, and DNA fragmentation (iii) ↓ levels of serum BUN, creatinine and LDH
[33]	Female Wistar albino rats (*n* = 18)	Spirulina was administered orally (1000 mg/kg bw/day) for 7 days, then a single dose of CP was injected i.p. (150 mg/kg) on the seventh day of the experiment.	(i) ↑ Tissue levels of SOD and CAT (ii) ↓ MDA levels and apoptosis (iii) The protective effect of spirrulina might be due to its antioxidant and antiapoptotic properties.
[34]	Male Wistar rats (*n* = 40)	Rats were pretreated with vitamin E (100 mg/kg) for 3 days and then, a single dose of CP (150 mg/kg, i.p) was administered.	(i) ↓ TNF-*α* and MDA production (ii) ↑ GPx and MDA levels in the renal tissues (iii) The protective effect might be due to the antioxidant activity of vitamin E.
[35]	Male Wistar rats (*n* = 36)	To induce nephrotoxicity, CP (150 mg/kg) was administered through i.p. route. *Murraya koenigii* extract (100 mg/kg and 200 mg/kg i.p.) was administered and oxidative stress parameters were measured.	(i) ↓ BUN, creatinine and LPO levels (ii) ↑ SOD, GSH levels (iii) *Murraya koenigii* extract showed protective effect against CP-induced nephrotoxicity
[36]	Male Swiss albino mice (*n* = 40)	Mice were treated with *Capparis spinosa* extract (CSE) orally in doses of 100, 200, and 400 mg/kg, for 5 consecutive days, and CP (200 mg/kg, i.p) was administered on the fifth day, 1 hour after the last dose of extract administration.	(i) ↓ BUN, creatinine and MDA levels. (ii) ↑ GSH levels (iii) Histological examination also confirmed the protective effect of CSE.
[37]	Male Swiss albino mice (*n* = 50)	Rats were pretreated with different doses of melatonin (MEL) (5, 10, and 20 mg/kg/day, i.p) for 5 days, then CP (200 mg/kg, i.p) was administered 1 hour after the last MEL on day 5.	(i) ↓ BUN and Creatinine levels (ii) ↓ MDA, PC, NO levels and MPO activity (iii) ↑ GSH, SOD, GPx and CAT activity (iv) Attenuated CP-induced histological changes of the kidney tissues
[38]	Male Swiss albino mice (*n* = 30)	Rats were treated with *Elaeagnus angustifolia* fruit extract (EAFE) orally at doses of 100, 200, and 400 mg/kg, respectively, for 5 consecutive days and CP (200 mg/kg, i.p.) on the 5th day, 1 hour after the last dose of the extract.	(i) ↓ MDA, creatinine, and BUN levels (ii) ↑ GSH level (iii) Histological evaluation of the kidneys also showed protective effect
[39]	Male BALB/c mice (*n* = 32)	Rats were administered with cerium oxide nanoparticles (NC) at a dose of 100 *μ*g/kg i.p. for 3 consecutive days. And, a single dose of CP (200 mg/kg, i.p) was administered on the third day. On the sixth day of the experiment, various parameters were evaluated.	(i) ↓ MDA, urea and creatinine levels• ↑ GSH level (ii) In addition, NC pre-treatment alleviated immunoreactivity of caspase-3and it showed strong antioxidant activity.
[40]	Male Parke's strain mice (*n* = 24)	CP (200 mg/kg, i.p) was administered (once in a week) for five weeks and also aqueous extract of *Phyllanthus fraternus* (AEPF) (400 mg/kg) for 5 weeks (once in a week) orally.	(i) ↑ SOD and CAT activity (ii) ↓ MDA levels (iii) Restored the percentage of the kidney somatic index
[41]	Male Wistar rats (*n* = 30)	Rats were treated with 100 and 200 mg/kg *Olea europaea* leaf extract (OLE) orally for 15 days and a single i.p injection of 150 mg/kg CP at day 16.	(i) ↓ BUN and creatinine levels (ii) ↑ Bcl-2 expression (iii) ↓ NF-*κ*B, Bax, cytochrome c and caspase-3 (iv) Upregulated Nrf2/ARE/HO-1 signaling (v) Enhanced the antioxidant activity, attenuating inflammation and apoptosis.
[42]	Male Swiss albino mice (*n* = 35)	Mice were pretreated with *Lavandula officinalis* L extract (LOE) orally at 100, 200, and 400 mg/kg for five consecutive days and CP (200 mg/kg, i.p.) was administrated on the fifth day, 1 hour after the last dose of the extract.	(i) ↓ MDA, BUN, and creatinine levels (ii) ↑ GSH, SOD, and CAT activity (iii) Histological examination of the kidneys also revealed the protective effect of LOE.
[43]	Male Sprague-Dawley rats (*n* = 42)	Rats were treated with carvacrol (CAR) at 5 and 10 mg/kg for 6 consecutive days, and CP (100 mg/kg) was administered at the fourth day.	(i) ↓ MDA, TOS, and OSI levels (ii) ↑ GSH, SOD, CAT, and TAC levels (iii) Lower tissue damage
[44]	Male Swiss Albino mice (*n* = 48, for each models)	To induce acute kidney injury, single dose of CP was administered at the dose of 75 mg/kg i.p., whereas the subacute kidney injury was induced by daily injection of CP (50 mg/kg i.p) for 1 week. Mice were treated with tranilast (300 mg/kg, orally) for 8 days in acute injury. In subacute kidney injury, mice were treated with tranilast for the first 7 days and then tranilast (300 mg/kg, orally) + CP (50 mg/kg, I.P.) for the successive 7 days.	(i) ↓ BUN, creatinine, TNF-*α*, LDH, total kidney protein contents, and lipid peroxidation (ii) ↑ SOD activity and GSH content (iii) This protective effect might be through enhancing antioxidant defense mechanisms, decreasing cytotoxicity, and decreasing expression of inflammatory cytokines.
[45]	Male albino Wistar rats (*n* = 35)	Rats were treated with *Mangifera indica* L. (MPS) (500, 1000 mg/kg, p.o.) and/or silymarin (150 mg/kg, p.o.) for 10 days. In the last 5 days of treatment, rats were administered CP (150 mg/kg, i.p).	(i) ↑ GSH and SOD activity (ii) ↓ Total MDA and GST (iii) The antioxidant effect of MPS and/or silymarin might be responsible for the kidney protection.
[46]	Male Sprague-Dawley rats (*n* = 66)	Rats were pretreated with single dose of Wuzhi capsule (WZC) (300 mg/kg), 15 minutes before receiving CP injection. One hour after the injections, all rats were injected with MESNA (420 mg/kg) to prevent possible bladder injuries.	(i) ↑ GSH, GPx, and SOD contents/or activities in both tissues and plasma (ii) ↓ BUN, creatinine, and MDA (iii) Improved morphology and pathology of the kidneys
[47]	Male Wistar albino rats (*n* = 40)	To induce renal toxicity, CP (200 mg/kg, i.p.) was administered as a single dose on first day of the experimental period, followed by the administration of taurine (200 mg/kg, i.p.) daily for 3 weeks.	(i) Serum activities of creatine kinase, creatine kinase isoenzyme, LDH, creatinine as well as BUN disturbances were attenuated (ii) CP-induced ECG changes were significantly reversed
[48]	Either sex Wistar rats (*n* = 30)	Rats were treated with daidzein, (20 and 40 mg/kg, p.o.) for 10 days and administered with CP (150 mg/kg, i.p.) in the last 5 days.	(i) ↓ MDA level (ii) ↑ GSH, SOD, and CAT levels (iii) Improved the structural architecture of renal profiles
[49]	Male Sprague-Dawley rats (*n* = 42)	Rats were administered with 0.5 or 1 mg/kg selenium for 6 consecutive days and then a single dose of CP (150 mg/kg, i.p.) was administered on the sixth day.	(i) Decreased creatinine levels in a dose-dependent manner. But, creatinine levels remained high relative to the control group, which indicates that selenium cotreatment might be partially effective. (ii) Cotreatment with selenium 1 mg/kg resulted in a better improvement of oxidative stress markers
[50]	Male Wistar albino rats (*n* = 35)	The rats were pretreated with chrysin (CH) orally in doses of 25 and 50 mg/kg for 7 consecutive days, and CP (200-mg/kg, i.p.) was administrated on the 7th day, 1 h after the last dose of CH.	(i) ↓ Urea, creatinine, MDA, and renal deterioration (ii) ↑ SOD, CAT, GPx, and GSH levels (iii) Furthermore, CH reversed the changes in levels of inflammatory, apoptotic, and autophagic parameters such as NF-*κ*B, TNF-*α*, IL-1*β*, IL-6, iNOS, COX-2, Bax, Bcl-2, and LC3B in kidney tissues.
[51]	Albino rats (*n* = 60)	Rats were pretreated with N-acetylcysteine (NAC) (10 mg/kg/d), melatonin (MT) (10 mg/kg/d), alpha-lipoic acid (ALA) (10 mg/kg/d) and MT + ALA, i.p. for 5 days before treatment with CP (150 mg/kg, i.p.) on day 5.	(i) ↓ Serum creatinine, urea, uric acid, potassium, sodium, chloride bicarbonate, and oxidative markers (ii) Combined administration of MT and ALA was found to be more effective
[52]	Male Swiss albino mice (*n* = 48)	Mice were administered with a single dose of CP (200 mg/kg, i.p.), and then followed by propolis (100 mg/kg) for 7 consecutive days.	(i) Improved the levels of urea and creatinine. (ii) Moreover, the histological picture of the kidney was significantly improved. (iii) In addition, propolis prevented liver toxicity and immunosuppression.
[53]	Male Wistar rats (*n* = 40)	Animals were pretreated with oral whey protein isolate (WPI) (75, 150 or 300 mg/kg/day), respectively, for 15 days before CP (200 mg/kg, i.p.) treatment on day 15.	(i) ↓ Renal MDA, NOx, MPO and IL-1*β* levels (ii) Treatment with WPI significantly protected against CP-induced damage showing marked dose-dependent antioxidant and anti-inflammatory properties.
[54]	Male albino mice (*n* = 40)	Hesperidin (HSN) was administered for 10 consecutive days at a dose of 100 and 200 mg/kg/day, orally. While, CP (200 mg/kg, i.p.) was administered on the fifth day, after starting HSN.	(i) ↓ Serum creatinine and cystatin C (ii) ↓ MDA, nitric oxide, Bax/Bcl-2 ratio, and caspase-3 levels (iii) HSN prevented CP-induced nephrotoxicity by tackling oxidative/nitrative stress, inflammation, and apoptosis.
[55]	Male albino rats (*n* = 46)	Rats were treated with fennel oil, an oil extracted from the seeds of *Foeniculum vulgare* (1 ml/kg, once a week for 6 weeks) orally. CP was administered orally at a dose of 15 mg/kg once a week for six weeks.	(i) ↓ creatinine, urea, PCNA, caspase-3, and Î±-SMA (ii) Improved the histological structure of the kidney (iii) In general, the ameliorative effect of fennel oil might be due to its antioxidant activity
[56]	Male Swiss albino mice (*n* = 36)	Iridoid glycosides enriched fraction (IGs), obtained from *Picrorhiza kurroa* was administered daily at 25, 50, and 100 mg/kg; orally for 21 days. Followed by CP (200 mg/kg, i.p.) intoxication for consecutive two days. To evaluate the role of PPAR-*γ* receptors for the protective effect of IGs, additional mice were pretreated with PPAR-*γ* antagonist (BADGE 5 mg/kg, i.p) followed by IGs (100 mg/kg; p.o.) for 21 days before CP intoxication.	(i) Treatment with IGs prevented renal tubular swelling, granular degeneration and glomerular damage. (ii) Improved the altered expressions of NF-kB, IL-1*β* and TNF-*α* (iii) The antiapoptotic effect of IGs was showed by the Bax/Bcl-2 expressions and caspase 3/9 activity in renal tissues. (iv) Improved the PPAR-*γ* expression in the kidney tissues
[57]	Male Sprague-Dawley rats (*n* = 24)	Rats were treated with boric acid (BA) for 6 days and CP (200 mg/kg) with BA (200 mg/kg) on the fourth day of the experiment.	(i) ↓ Serum creatinine, BUN, MDA, and NO levels (ii) ↑ CAT, GSH, and GPx levels (iii) BA-induced renoprotection might be due to an increase in the activity of the antioxidant protection system and also inhibition of lipid peroxidation.
[58]	Sprague-Dawley rats (*n* = 18)	N-acetylcysteine (NAC) (100 mg/Kg) was administered i.p., once daily for 5 consecutive days and followed by a single dose of CP (200 mg/Kg), 1 hour after the last dose.	(i) NAC re-established the GSH pool and preserved the normal histoarchitecture of the kidney. (ii) This might be due the antioxidant properties of NAC.
[59]	Male Wistar rats (*n* = 33)	Animals were treated with *Echinodorus macrophyllus* (ECM) (2 g/kg) by oral gavage once a day for 5 days, and followed by single dose of CP (150 mg/kg, i.p.), in the fifth day of the experiment.	(i) ↑ creatinine clearance and levels of thiol in the kidney tissue (ii) ↓ Excretion of urinary peroxides (iii) ↓ TBARS
[60]	Male albino rats (*n* = 20)	Rats were coadministered orally with CP (20 mg/kg) and aqueous leaf extract of *Acalypha wilkesiana* (110, 220, and 440 mg/kg) leaf extract daily for 7 days.	(i) ↓ Plasma creatinine, urea, and uric acid (ii) ↑ Plasma SOD, CAT, GST, and the level of GSH in a dose-dependent manner.
[61]	Male Swiss albino mice (*n* = 30)	Rats were treated with *Eucalyptus globulus* (EG) (50 and 100 mg/kg) once daily for 15 days along with CP (200 mg/kg, on 12th and 13th day). All treatments were administered intraperitoneally.	(i) ↓ creatinine, BUN, and liver enzymes. (ii) ↓ MDA levels (iii) ↑ GSH contents (iv) This protective effect might be partially through induction of Nrf2/HO-1 signaling with attenuation of excessive inflammatory responses as well as apoptosis in renal tissues.
[62]	Male Wistar rats (*n* = 5–7 rats per group, 4 groups in total)	Rats were pretreated with aminoguanidine (AG) at a dose of 200 mg/kg i.p. 1 hour before the administration of CP at a dose of 150 mg/kg.	(i) AG prevented lipid peroxidation, protein oxidation, depletion of reduced GSH, and loss of activities of the antioxidant enzymes, including GPx, catalase, and GSTase and also MPO activity. (ii) This effect might be through inhibiting oxidative stress.
[63]	Either sex of SD rats (*n* = 66)	Nephrotoxicity was induced with a single administration of CP 200 mg/kg, i.p., on the first day. Then, followed by the treatment of *Croton macrostachyus* (CM) crude extract and solvent fractions orally for 10 days.	(i) ↓ Serum creatinine and BUN (ii) Histopathological results also confirmed the protective effect of the crude extract and solvent fractions of CM.
[64]	Male ICR mice (*n* = 40)	Animals were treated once daily with CP (80 mg/kg/day) for 5 days and pyrroloquinoline quinone (PQQ) (5, 10, and 20 mg/kg/day) for another 14 days.	(i) ↓ Serum levels of creatinine and urea (ii) ↓ MDA, IL-1*β*, IL-6, and TNF-*α* levels (iii) PQQ prevented nephrotoxicity probably by activating the Nrf2-mediated antioxidant pathway and through inhibiting the NLRP3 inflammatory pathway.
[65]	Male swiss albino mice (*n* = 30)	Animals were treated with rutin (40 mg/kg and 80 mg/kg) after the administration of CP (25 mg/kg), respectively, for 14 days.	(i) Rutin treatment significantly reversed the status serum biomarkers, hematological variables, and antioxidant markers
[66]	Male SD rats (*n* = 70)	Rats were administered with single injection of CP (150 mg/Kg, i.p) and followed by garcinol treatment (10 mg/Kg, orally/daily) for 4 weeks.	(i) ↓ IL-1b, IL-6, monocyte chemotactic protein-1(MCP-1), macrophage inducible c type lectin (mincle), spleen tyrosine kinase (Syk), transcriptional factor (NF-kB), and toll-like receptor (TLR-4). (ii) This protective effect might be through inhibiting mincle expression, and prevents Syk and TLR-4/NF-kB activation.
[67]	Male albino rats (*n* = 18)	Animals were intraperitoneally injected with a single dose of CP (200 mg/kg) and oral administration of *Sargassum cinereum* extract at dose of 180 mg/kg for consecutive 20 days.	(i) Ameliorated hematological, biochemical, oxidative damages and histopathological changes induced by CP-injection.
[68]	Female Balb/c mice (*n* = 90)	Mice were administered with CP (25 mg/kg, i.p.) for 10 consecutive days and *Pithecellobium dulce* fruit extract (P. dulce) (40 mg/kg) for 10 consecutive days orally, starting from the same day of CP administration.	(i) ↓ Serum urea and creatinine levels. (ii) CP-induced immunosuppression accompanied with urotoxicity, hepatotoxicity, and nephrotoxicity were ameliorated.
[41]	Male Wistar rats (*n* = 30)	Rats were treated with Olive leaf extract (OLE) 100 or 200 mg/kg body weight for 15 days and a single injection of 150 mg/kg CP at day 16.	(i) ↑ antioxidant defenses and Bcl-2 expression (ii) ↓ Proinflammatory and proapoptotic markers NF-*κ*B, Bax, cytochrome c, and caspase-3. (ii) ↑ Nrf2/ARE/HO-1 signaling, enhanced the antioxidant activity and attenuated inflammation and apoptosis.
[69]	Male albino Wistar rats (*n* = 24)	Rats were pretreated with Formononetin (FOR) (40 mg/kg/day) 15 days followed by CP-injection on the 16th day.	(i) Enhanced the level of antioxidants and suppressed oxidative stress (ii) Proinflammatory mediators and apoptosis were also suppressed due to FOR treatment. (iii) Protective effect might be due to attenuation of the oxidative damage and inflammation.

Tumor necrosis factor-*α* (TNF-*α*), nuclear factor kappa B (NF-*κ*B), interleukin-6 (IL-6), interleukin-1*β* (IL-1*β*), inducible nitric oxide synthase (iNOS), cyclooxygenase-2 (COX-2), light chain 3B (LC3B), malondialdehyde (MDA), number of animals used (n), gluthathione (GSH), superoxide dismutase (SOD), catalase (CAT), terminal deoxynucleotidyl transferase-mediated dUTP-biotin Nick end labeling assay (TUNEL), glutathion peroxidase (GPx), protein carbonyl (PC), total oxidant state (TOS), oxidative stress indexes (OSI), total antioxidant capacity (TAC), and glutathione-S-transferase (GST).

**Table 2:** The effect of currently available drugs against cyclophosphamide-induced nephrotoxicity.

Reference	Animal used	Method and intervention	Major findings
[70]	Male albino rats (*n* = 24)	Animals were treated with Tolvaptan (TOL) 10 mg/kg/d, orally for 22 days with concomitant administration of CP 75 mg/kg i.p. on days 3, 4, 5, 19, 20, and 21 of the experiment.	Treatment with TOL resulted in significant improvement in the level of urine volume, urinary creatinine, and significant reduction of body weight, serum creatinine, urea, serum potassium, urine osmolarity. TOL resulted significant reduction of blood pressure and offered protection to the heart and kidney. TOL administration significantly decreased the level of caspase-3 and Bax with increased expression of antiapoptotic Bcl-2 in renal tissue.
[20]	Male Wistar rats (*n* = 32)	Animals were treated with Alogliptin (ALO) 20 mg/kg/day; p.o. for 7 days with single CP 200 mg/kg; i.p. injection on day 2.	Treatment with ALO declined serum kidney function markers, oxidative stress and apoptosis markers, MAP3K expression, phospho (p)-SMAD3, p-JNK, and p-c-Jun levels.

Alogliptin (ALO), Cyclophosphamide (CP), decapentaplegic homolog 3 (SMAD3), c-Jun N-terminal kinase (JNK), mitogen-activated protein kinase (MAPK), number of animals used (n), and Tolvaptan (TOL).

## Abbreviations

CAT: Catalase
CP: Cyclophosphamide
CVDs: Cardiovascular diseases
GSH: Glutathione
GSH-Px: Glutathione peroxidase
KIM-1: Kidney injury molecule-1
NGAL: Neutrophil gelatinase-associated lipocalin
ROS: Reactive oxygen species.
